# Fabrication of covalently linked exfoliated boron nitride nanosheet/multi-walled carbon nanotube hybrid particles for thermal conductive composite materials†

**DOI:** 10.1039/c8ra05620j

**Published:** 2018-10-01

**Authors:** Kiho Kim, Hyunwoo Oh, Jooheon Kim

**Affiliations:** School of Chemical Engineering & Materials Science, Chung-Ang University Seoul 06974 Republic of Korea jooheonkim@cau.ac.kr

## Abstract

Boron nitride nanosheet (BNNS)/multi-walled carbon nanotube (MWCNT) hybrid particles were synthesized for use as a conductive filler for epoxy and polyphenylene sulfide (PPS). BNNSs were prepared *via* the exfoliation of bulk boron nitride (BN) particles. Micrometer-sized BN particles were exfoliated to form nanosheets, and their surfaces were modified using 3-aminopropyltriethoxysilane (APTES). The amine groups on the BNNS surface were reacted with acid-treated MWCNTs, and covalently connected BNNS/MWCNT particles were synthesized. Moreover, a chemical reaction without agitation increased the particle connection during the hybrid particle preparation, resulting in a large number of MWCNTs being introduced onto the BNNSs. The BNNS/MWCNT hybrid particle composite had better thermal conductivity than BNNSs or a BNNS/MWCNT composite without chemical bonding based on the same filler contents and composition. This was because of the particle connections establishing three-dimensional heat conducting path in a matrix, which affected the thermal conductivity of the composite.

## Abstract 2

Boron nitride nanosheet (BNNS)/multi-walled carbon nanotube (MWCNT) hybrid particles were synthesized for use as a conductive filler for epoxy and polyphenylene sulfide (PPS).

## Graphical abstract

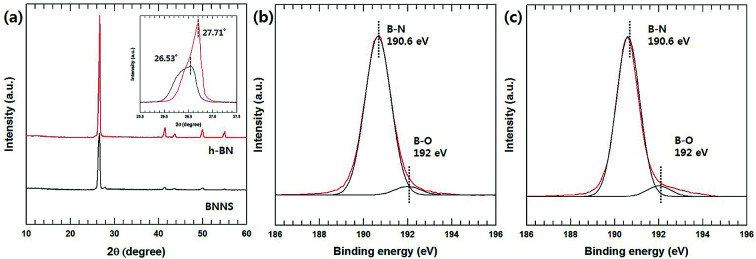

## Introduction

Polymer-based materials with high thermal conductivity are in constant demand to ensure the life span and performance of various devices such as electronics, automobiles, and lighting equipment.^1,2^ In particular, the life span of the photoluminescence of light emitting diodes is considerably influenced by their operating temperature. Therefore, it is essential that the heat be dissipated as quickly and as effectively as possible to maintain the desired device operating temperature.^3–5^ Traditionally, thermal issues in encapsulated devices have been addressed using embedded heat sinks, which are often susceptible to thermal cracking and have limited utility in thin devices. Accordingly, polymers with thermally conductive fillers are emerging as cost-effective ways of addressing thermal management issues.^6,7^

Heat conduction is mainly comprised of two mechanisms. The first is phonon transfer. A phonon is an acoustic vibration that is easily transferred in highly crystalline materials such as ceramics.^8^ The other mechanism is heat conduction *via* electrons, which dominantly occurs in metals or some carbonaceous materials like graphene and carbon nanotubes.^9^ Unfortunately, most polymers have an amorphous structure or low crystallinity with an electrically insulating property, which results in a low thermal conductivity (∼0.3 W m^−1^ K^−1^). Some researchers have developed polymers with high thermal conductivity using oriented mesogen structures to obtain high crystallinity and thermal conductivity. However, those highly crystalline polymers have a high viscosity and cost, which reduce their processability and applicability.^10,11^ Therefore, most studies have focused on composite materials that use fillers with high thermal conductivities. Traditionally, metal particles and carbonaceous particles have been used as fillers. Nowadays, ceramic particles are widely applied because of their outstanding thermal conductivity, relatively low density, chemical stability, and electrical insulation property. To achieve high thermal conductivity, a heat flow path should be generated using a connected, thermally conductive filler, which is known as percolation threshold. There are several approaches for maximizing particle connections such as the structure control, use of a compound containing several types of fillers or filler sizes.^12–14^ Among these approaches, a ceramic/carbonaceous hybrid system is commonly adopted because flexible carbonaceous particles provide interconnections between the ceramic filler particles, which generate thermally conductive bridges. In particular, the one-dimensional, structured multi-walled carbon nanotube (MWCNT) is a promising material for a secondary filler because of its ultra-high thermal conductivity and small size.^15,16^ Unfortunately, MWCNTs are easily aggregated and entangled with each other, making a uniform dispersion in a polymer matrix is an important issue. To achieve highly dispersed MWCNTs, surface modification is widely applied to introduce polar functional groups or coupling agents compatible with the polymer matrix. The surface-modified MWCNTs can then be homogeneously dispersed in the polymer matrix, which increases the probability of physical connections with the ceramic filler.^17,18^ On the other hand, to maximize the particle connections, some studies have focused on the fabrication of hybrid particles *via* the growth of MWCNTs on ceramic particles using chemical vapor deposition. This method could fabricate well-connected hybrid particles but required a highly processed coating, and only a very small amount of product was fabricated in the experiments.^19,20^

In this study, boron nitride was adopted as the thermally conductive ceramic filler, because it has the highest thermal conductivity of electrically insulating materials. In addition, hybrid particles composed of boron nitride nanosheets (BNNSs) and MWCNTs were fabricated as a thermally conductive filler *via* the chemical bonding of these particles. The boron nitride (BN) particles were exfoliated to form BNNSs through a water steam treatment using a humidifier and tubular furnace because BNNSs have better thermal conductivity than bulk BN. To chemically connect the BNNSs and MWCNTs, it was necessary to provide an appropriate surface modification and coupling agent that could reach both ends of each particle. Therefore, the BNNSs were fabricated *via* the exfoliation of BN particles, and their surfaces were modified using aminopropyltriethoxysilane (APTES). The MWCNTs were functionalized *via* acid treatment to introduce the carboxylic group onto the MWCNT surface. The APTES-treated BNNSs (BNNS-NH_2_) and MWCNTs with the introduced carboxylic group (MWCNT-COOH) were chemically reacted *via* two different methods. The first was a simple solvent process, which was performed using particle suspension with agitating. The other method was a vacuum-filtration-supported method (VFSM). The BNNS-NH_2_ and MWCNT-COOH were stacked *via* the vacuum filtration of the suspension, resulting in a stacked particle mixture that was chemically connected using a catalyst and heat treatment. The amounts of MWCNTs introduced with these methods were compared. The thermal conductivities of the composites were also compared based on the filler composition and loading.

## Experimental

### Materials

Epoxy-terminated dimethyl siloxane (ETDS) was purchased from Shin-Etsu Silicon (KF-105, epoxide equivalent weight (EEW) = 166.6 g per eq., density = 1.20 g cm^−3^) and used after drying completely under vacuum at 50 °C for 24 h. 4,4′-Diaminodiphenylmethane (DDM), manufactured by TCI Korea, was used as the curing agent without further purification. The MWCNTs were received from LG Electronics in Korea. The BN was purchased from ESK Ceramics/3M in Germany. *N*,*N*-Dimethylmethanamide (DMF, Samchun Chem., Korea) was sued without further purification. 3-Aminopropyltriethoxysilane (APTES) and *N*,*N*′-dicyclohexylcarbodiimide (DCC) were purchased from Sigma-Aldrich Korea.

### Preparation of BNNSs

The BNNSs were prepared by the exfoliation of micrometer-scale BN powder. The BN powder was loaded in a ceramic boat and placed into the hot zone of a horizontal tubular furnace. Deionized (DI) water was ultrasonically sprayed using a home humidifier (60 MHz and 35 W) and carried by Ar gas at a flow rate of 200 mL min^−1^. The temperature of the furnace was kept at 850 °C for 2 h. Then, the furnace was cooled down to room temperature, and the hydroxylated BN (BNO) was collected. After hydroxylation, the 0.5 g of BNO was dispersed in 40 mL of DI water by sonication for 2 h for the exfoliation of the BN layer and then left without sonication for 1 h. This allowed the BN powder to settle to the bottom, and a milky suspension could be separated. The BNNSs were obtained *via* the vacuum filtration of this milky suspension.

### Fabrication of chemically bonded BN-MWCNT particles

The BNNSs were chemically modified using a silane coupling agent, APTES. The APTES (3 wt%) was added to DI water and ethanol (7 : 3 mixture) and stirred at 50 °C for 30 min to achieve hydrolysis. The as-prepared particles were added to the above solution and stirred at 80 °C for 12 h. In this reaction, oxalic acid was used as a catalyst. The resulting particles (BNNS-NH_2_) were then rinsed with DI water, filtered three times, and dried in a convection oven at 80 °C for 5 h to remove the solvent.

Next, the MWCNTs were purified and functionalized with carboxylic acid functional groups by heat treating the MWCNTs (4 g) in 400 mL of H_2_SO_4_ and HNO_3_ (3 : 1 by volume) in an ultrasonicator bath for 2 h at room temperature to form a suspension. The suspension was then heated to 80 °C and stirred for 1 h. The acid treatment produced carboxylic groups on the surfaces of the MWCNTs without large decreases in the MWCNT length. This reaction also led to the elimination of impurities using DI water and centrifugation. Finally, the carboxylic functionalized MWCNTs (MWCNT-COOH) were dried in a convection oven.

**Scheme 1:**
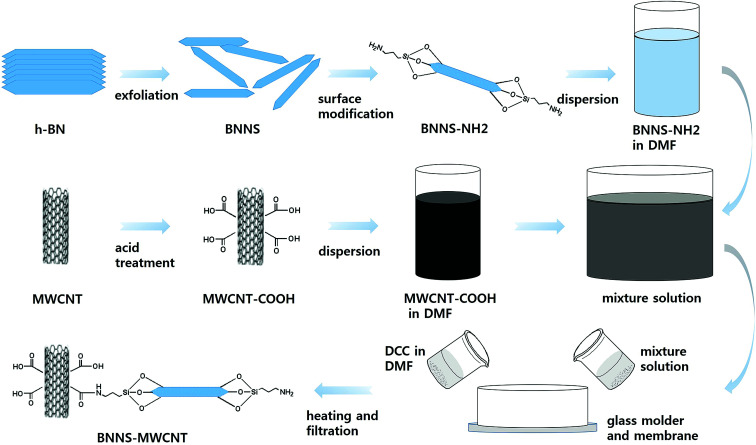
Fabrication procedure of BNNS-MWCNT hybrid particle *via* VFSM.

The BNNS-MWNCT hybrid particles were fabricated *via* two different methods. The first method was a simple solution process (SP). The BNNSs and MWCNT-COOH were suspended in DMF. Then, 1 g of DCC was added to the suspension and stirred mildly for 2 h at 80 °C. The other method was a vacuum-filtration-supported method (VFSM). The BNNS/MWCNT-COOH/DMF solution was vacuum filtrated using a silicon oxide membrane (0.2 μm, Anodisc, Whatman, USA) and DCC dissolved DMF was poured into a glass mold with very slow suction. In this step, the glass mold was heated to 80 °C using a custom-made heat source wrapped around the glass mold. Finally, the fabricated hybrid particles were rinsed with acetone several times to remove unreacted MWCNT and dried in a convection oven.^21^ The particle fabrication method VFSM were summarized in Scheme 1.

### Fabrication of ETDS matrix and composites

Thermoset and thermoplastic-based composites were fabricated using ETDS resin and PPS, respectively. A mixture of ETDS and DDM was prepared with a 1 : 2 equivalent weight ratio. DDM (1.9 g) and ETDS resin (9.5 g) mixture was heated in an oil bath at 120 °C for under a N_2_ atmosphere until opaque mixture turn to yellowish transparent liquid. The bubbles in the mixture were removed by placing the flask in a vacuum oven for 30 min at room temperature. The ETDS-based composites were prepared by solution blending and a casting method. The fillers were added to the epoxy resin for approximately 1 h with a minimum amount of ethanol until the synthesized materials were completely mixed. Composite films were fabricated to uniform thickness *via* a doctor blade on the Teflon mold, these composite films were pre-cured at 80 °C for 3 h until no air bubbles appeared on the surface in vacuum oven, followed by post-curing at 180 °C for 5 h.

The melt mixing of the PPS composites was performed in a twin extruder (model BA-11, *L*/*D* ratio = 40, Bau Technology) at a specified temperature range. The temperatures of the feeding zone, melting zone, mixing zone, and exit die were 270, 280, 290, and 300 °C, respectively. The feeding rate of the materials and the extrusion speed were held constant at 100 g min^−1^ and 300 rpm, respectively. The melt-mixed composites were immediately quenched in a water bath after extrusion. The composites were dried in an air-circulating oven at 80 °C for one day before use. Specimens for thermal diffusivity tests were prepared using a mini-injection molder (DSM Xplore, Micro-injection Moulding Machine 5.5 mL).

### Characterization

The fabricated BNNS, MWCNT-COOH and BNNS-MWCNT were characterized by X-ray photoelectron spectroscopy (XPS, Thermo U.K. Kalpha) using an Al K_α_ X-ray source (1486.6 eV) and a hemispherical analyzer. During curve fitting, the Gaussian peak widths were constant in each spectrum. The characterization of the crystal structure of the BN was performed using an X-ray diffractometer (XRD, Bruker-AXS, New D8-Advance) at a scan rate of 1° s^−1^ with a 2*θ* range of 10–70° with Cu K_α1_ radiation (*λ* = 0.15406 nm). Thermogravimetric analyses (TGA; TGA-2050, TA Instruments) of the samples were carried out to examine the thermal degradation process, with 4 mg samples heated to 800 °C at a heating rate of 10 °C min^−1^ under an air atmosphere. Field emission scanning electron microscopy (FE-SEM, Sigma, Carl Zeiss), and high resolution transmission electron microscopy (HR-TEM, JEM-3010) were used to examine the morphology of the fabricated particles and composites. The thermal transport performance of the fabricated composite was characterized by laser flash analysis (LFA, Netzsch Instruments Co, Nanoflash LFA447) and differential scanning calorimetry (DSC, Perkin-Elmer Inc., DSC-7) at room temperature. The transferred signal initiated a thermal equilibration process in the composite specimen, which was recorded using a difference detector at the rear surface and used to evaluate the thermal diffusivity. The bulk density *ρ*_comp_ (g cm^−3^) of each specimen was measured using the Archimedes water displacement method. The thermal conductivity (*k*) was calculated by multiplying the thermal diffusivity, density, and specific heat capacity of the composite.

## Results and discussion

**Fig. 1:**
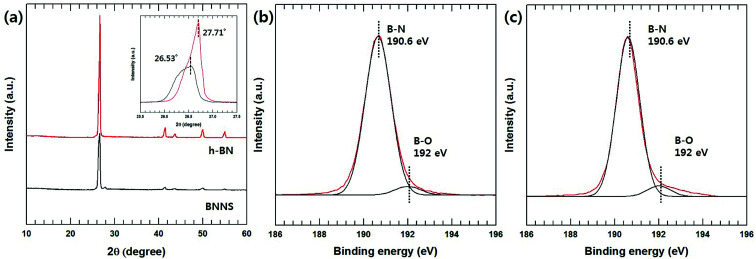
XRD and XPS analysis of h-BN and BNNS. (a) XRD pattern of h-BN and BNNS (inset: high magnification of 002 peaks), (b) XPS B1s spectra of h-BN, and (c) XPS B1s spectra of BNNS.

The chemical and morphological structures of the BNNSs are compared with those of h-BN particles in Fig. S1.†Fig. 1(a) shows the XRD patterns of h-BN and BNNS. The XRD pattern of the h-BN powder reveals several diffraction peaks centered at 26.6°, 41.7°, 44.0°, 50.2° and 55.1°, corresponding to the (002), (100), (101), (102), and (004) planes, respectively.^22^ The XRD pattern of the BNNSs was similar to that of h-BN, except for a new peak at 27.9°. This new peak was due to the (010) B(OH)_3_ diffraction, indicating the formation of hydroxyl surface groups.^23^ Moreover, BNNS results show that the intensity of the (002) peak significantly decreases and the two-theta peak slightly downshifts from 26.7° to 26.53°, which was evidence of an increase in the interplanar distance *via* exfoliation. The increased interplanar distance and decreased intensity of the other diffraction peaks [(100), (101), (102), (004)] suggest the formation of ultrathin h-BN sheets with a less-extended/ordered stacking in the *c* direction.^23–25^ Moreover, the chemical difference was also confirmed *via* the XPS analysis results shown in Fig. 1(b) and (c). The B1s peaks of the h-BN particles consisted mainly of the B–N peak at 190.6 eV with a very weak one at 192 eV for the corresponding B–O bonding. On the other hand, a stronger B–O peak was generated for the BNNSs, which originated with the hydroxyl groups resulting from the BN hydroxylation reaction during the heating process. These hydroxyl groups caused a repulsion force and the exfoliation of each BN layer *via* sonication.

**Fig. 2:**
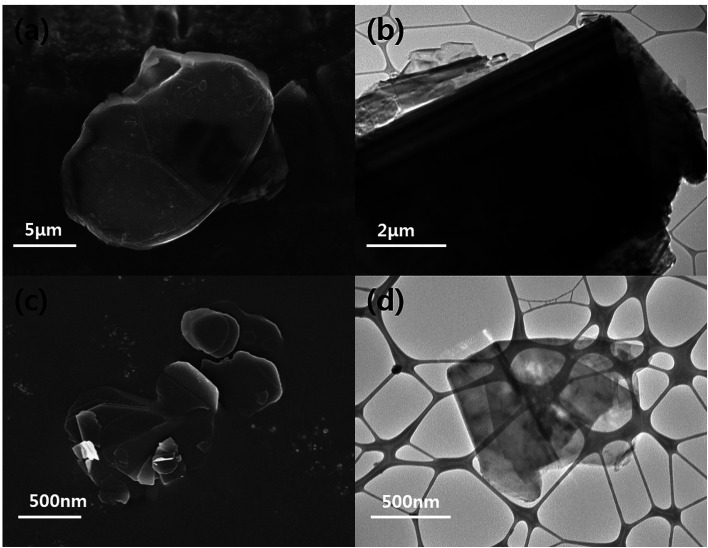
FE-SEM and HRTEM images of h-BN and BNNS. (a) FE-SEM image of h-BN, (b) HR-TEM image of h-BN, (c) FE-SEM image of BNNS and (d) HR-TEM image of BNNS.

The particle morphologies of the h-BN and BNNSs were observed by FE-SEM and HR-TEM and are shown in Fig. 2(a)–(d). Pristine h-BN presents stackable sheet structures with dimensions on the micrometer scale, along with smooth surfaces and edges. After the reaction with water and exfoliation, compared to the bulk h-BN powder, the BNNSs demonstrate a much smaller size and nanosheet-like morphology. Similar results were also obtained by HR-TEM: the h-BN had an opaque structure and a micrometer-scale thickness. On the other hand, the BNNSs had an almost transparent image, which meant they had an ultrathin structure because of the exfoliation. The size and thickness of BNNS were confirmed *via* AFM in Fig. S1.†

In this study, APTES was introduced onto the BNNS surface to assign amine groups that could chemically react with other nanomaterials. The preparation of silane coupling modified BN particles was reported several times in our previous studies, corresponding analysis did not contain in this manuscript.^26–28^ Moreover, an acid treatment using sulfuric acid and nitric acid is also a well-known method for functionalizing MWCNTs. However, it was required to compare with BNNW-MWCNT to confirm chemical bonding with BNNS and MWCNT. Therefore, XPS data of the MWCNT functionalization are contained in the ESI (Fig. S2†).

**Fig. 3:**
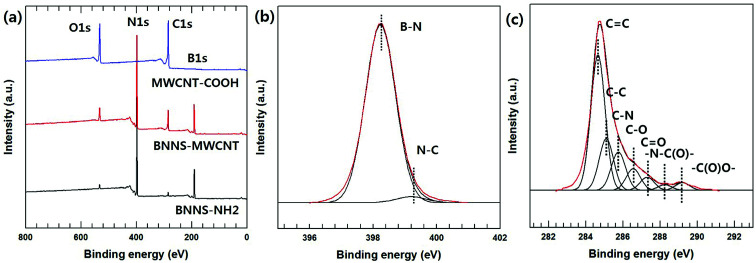
XPS analysis of fabricated BNNS-MWCNT hybrid particle. (a) XPS wide scan spectra of BNNS-NH_2_, MWCNT-COOH, and BNNS-MWCNT, (b) N1s spectra of BNNS-MWCNT, and (c) C1s spectra of BNNS-MWCNT.

**Fig. 4:**
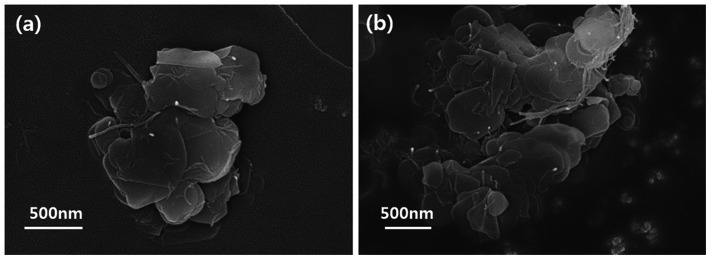
FE-SEM images of prepared BNNS-MWCNT hybrid particles. (a) BNNS-MWCNT *via* SP, and (b) BNNS-MWNCT *via* VFSM.

The chemical bonding of the BNNS-MWCNT hybrid particles fabricated *via* VFSM was confirmed using XPS analyses. Fig. 3(a) shows wide scans of the pristine BNNS-NH_2_, MWCNT-COOH, and BNNS-MWCNT, as well as the fits of the N1s and C1s peaks of the BNNS-MWCNT. As expected, the hybrid particles showing atomic peaks for both the BNNS-NH_2_ and MWCNT-COOH. However, these results cannot clearly verity the chemical bonding of two particles. In this hybrid particle, de-convoluted N1s peak could demonstrate the chemical bonding accurately due to amine group on the silane coupling agent were converted to amide bonding after reaction as shown in Scheme 1. Therefore, N1s deconvolution was performed and its result was shown in Fig. 3(b). The N1s peaks of BNNS-MWCNT were fitted to two component peaks. The lower binding energy peak at 398.2 eV was assigned to B–N bond associated with BNNS particle. The higher binding energy peak at 399.2 eV was assigned to N–C bonding. Unfortunately, the chemical bonding cannot confirm using this result because the signal of B–N bonding is too strong, other peaks were almost not distinguishable, resulting in this result did not have reliability. Therefore, C1s peak was de-convoluted and their result was shown in Fig. 3(c). The C1s peak was de-convoluted into seven binding energies at 284.72 (C=C), 285.1 (C–C), 285.7 (C–N), 286.2 (C–O), 287.5 (C=O), 287.9 (–N–C(O)–) and 289.2 eV (–C(O)O–). Especially, –N–C(O)– bonding was attributed to the formation of amide bonds between the carboxylic carbon and the primary amine of the silane coupling agent.^29–31^ These results are in good agreement with the reactions that occurred between the functionalized MWCNT and BNNS particles. The morphologies of the BNNS-MWCNT particles provided further evidence that the functionalized MWCNTs were successfully doped onto edge of the amine-functionalized BNNSs (Fig. 4 and Fig. S3†). It can be confirmed that the much amount of MWCNTs were introduced onto BNNS surface *via* VFSM than SP. The XPS spectra of the BNNS-MWCNT particles produced using the two types of experimental methods are also shown in Fig. S4.† As shown in Fig. S4,† the amount of introduced MWCNTs could be compared to the intensity of amide peak. As shown, ratio amide of the VFSM BNNS-MWCNT particles was much higher than that of the particles produced using SP method. This shows that VFSM was a more effective fabrication method than the conventional method. Sufficient contact with the reaction site is essential to achieve chemical bonding. To uniformly disperse the filler, the amount of filler is restricted and few reaction sites are used for the total suspension. Moreover, because of the continuous agitation of the suspension, the contact of each functional group should be disturbed. On the other hand, VFSM should not only maximize the particle connections, but also easily control the particle contact period. As a result, a much larger number of MWCNTs were introduced onto the BNNSs.

**Fig. 5:**
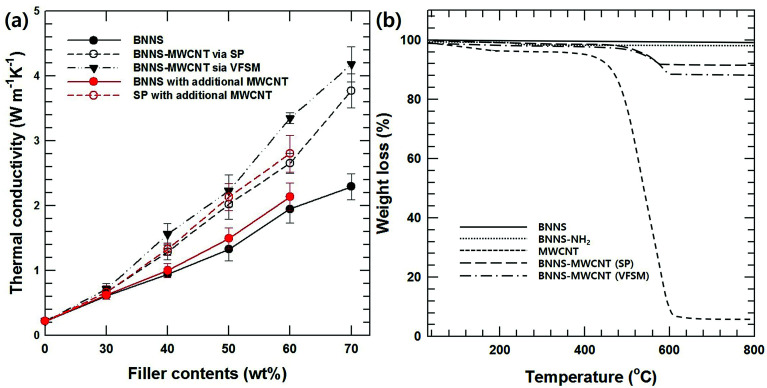
Thermal conductivity and TGA results of various BNNS-MWCNT composite and particles. (a) BNNS composite and BNNS-MWCNT composites with two kinds of fabrication method. The red lines are MWCNT contents calibrated composites for accurate thermal conductivity comparison. (b) TGA of prepared particles.

Additional MWCNTs produce a higher thermal conductivity than inorganic/polymer composite materials because they form a three-dimensional heat conducting network in the matrix, which is characterized by a high aspect ratio.^17,18^Fig. 5(a) shows the thermal conductivities of composites with BNNS and the two types of BNNS-MWCNT hybrid particles according to the fabrication methods that were examined as a function of the filler contents. The thermal conductivities of all the composites increased with increasing filler loading. Moreover, the MWCNT-introduced BNNS composite showed a higher thermal conductivity than BNNS composite. In particular, VFSM showed an outstanding thermal property compared to not only BNNS but also SP based on the same filler loading. This difference between two kinds of hybrid particles originated from the BNNS and MWCNT composition differences in the fabrication methods, which are shown in Fig. 4. Therefore, an arithmetical calculation of the filler content is meaningless. To make a more specific comparison of the effects of the hybrid particle fabrication methods, it is essential to modify the BNNS and MWCNT ratio using additional MWCNTs.

Fig. 5(b) shows the TGA results for BNNS, BNNS-NH_2_, MWCNT-COOH, and the two kinds of BNNS-MWCNT particles to analyze the BNNS/MWCNT ratio. Under the analysis conditions, BNNS-NH_2_ and MWCNT-COOH showed weight losses of 2.1 and 92.6%, which were derived from thermal degradation of the silane coupling agent and MWCNTs. Based on these results, the BNNS/MWCNT ratios of the two kinds of hybrid particles were 10.9 and 6.7, respectively.

**Fig. 6:**
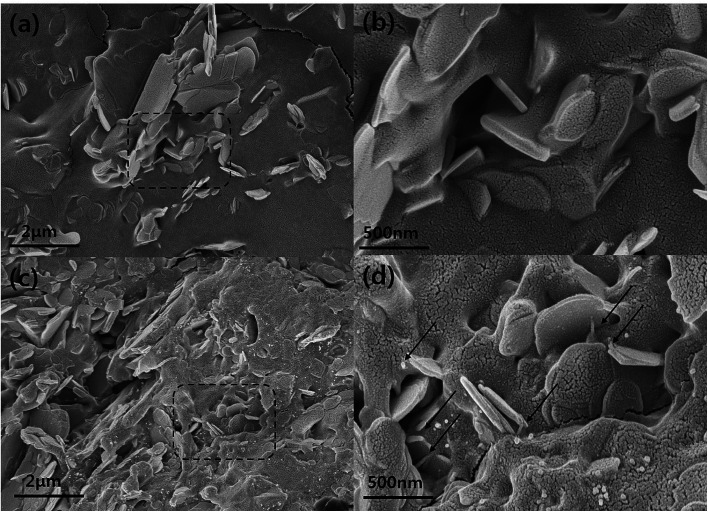
Cross sectional FE-SEM images of epoxy composites with BNNS and BNNS-MWCNT fillers with 30 wt% loading. (a) Low magnification FE-SEM image of BNNS composite, (b) high magnification FE-SEM image of h-BN composite, (c) low magnification FE-SEM image of BNNS-MWCNT composite, and (d) high magnification FE-SEM image of BNNS-MWCN composite (denoted arrows are air MWCNTs).

Based on TGA results, excess MWCNT-COOH was added to SP and BNNS composites during the composite fabrication for an accurate comparison with the same BNNS/MWCNT ratio and effect of chemical bonding. Fig. 5(a) also shows the thermal conductivities of the non-bonded BNNS, SP with additional MWCNT-COOH, and VFSM composites as a function of the filler content. Unfortunately, 70 wt% of BNNS and SP composite with additional MWCNT-COOH could not prepared due to too high viscosity. As expected, on the other hand, the chemically bonded BNNS-MWCNT filler composite shows the highest thermal conductivity, and VFSM also has the most outstanding performance at the whole filler concentration. These results were strong evidence for chemical reaction between BNNS and MWCNTs were positively influenced on thermal conductivity. Many previous studies have reported that the role of MWCNTs in a ceramic/polymer composite is connecting the ceramic particles. However, MWCNTs without ceramic fillers only cause a small increase in thermal conductivity despite their ultra-high thermal conductivity because of their extremely small particle size and high surface energy cause the twisted and entangled bundle. These MWCNT bundles lead the smaller mean particle size and much amount of phonon scattering at the surface, resulting in thermal conductivity was decreased without forming the effective thermal conductive path. Therefore, it is essential to form a BNNS/MWCNT contact for thermal conductivity enhancement effect *via* additional MWCNTS. Likewise, the particle connection probability was very low for the non-bonded BNNS and MWCNTs, especially at a low filler concentration. On the other hand, the already chemically bonded BNNS-MWCNT particles easily connected with each other, resulting in MWNCTs generate heat flow bridge between the BNNSs and enhanced the higher thermal conductivity. Fig. 6 presents the cross-sectional morphologies of the composites with 30 wt% of fillers. The non-covalent bonded particles were randomly dispersed and only a few connections and bridges were observed.^32,33^ After the covalent bonding, a much larger number of MWCNTs were connected to BNNSs, and these MWCNTs were easily connected to other particles. These results are in good agreement with the thermal conductivity behaviors.

Table 1 summarized the related previously reported thermal conductive composites for exfoliated BNNSs and hybrid particle. The various works reported that the hybrid particle system enhanced the thermal conductivity. Wu *et al.* fabricated the BNNS and surface modified BNNS to increase the heat flow region. The good dispersion and large surface area of surface modified BNNS promote the diffusion of phonon in composite.^34^ Pak *et al.* reported that the synergetic effect was strongly depended on surface modification of MWCNTs due to their dispersion.^35^ However, the fabrication of BNNS-MWCNT hybrid particle did not previously reported and this hybrid particle effectively enhanced the thermal conductivity without any specific fabrication method such as segregated structure and ice-templated assembly.^36,37^

**Table 1:** Through-plane thermal conductivity of BNNS and hybrid filler composites

Filler	Matrix	TC of matrix [W m^−1^ K^−1^]	TC of composite [W m^−1^ K^−1^]	Filler fraction	Method	Ref.
BNNS	Epoxy	0.25	1.63	25.1 vol%	Simple casting	38
BN, CNT	PVDF	0.23	1.913	30 vol%, 1 vol%	Melt mixing	39
Graphene, BNNS	PS	0.155	0.662	20 wt%, 1.5 wt%	Hot pressing	40
Modified BNNS	SBR	0.16	0.57	10.5 vol%	Slurry compounding	34
BN, modified MWCNT	PPS	0.31	1.74	50 wt%, 1 wt%	Melt mixing	35
Modified BN, modified MWCNT	Epoxy	0.18	1.91	30 vol%, 1 vol%	Simple casting	41
BN, MWCNT	UHMWPE	0.4591	1.794	49 wt%, 1 wt%	Segregated structure	36
BNNS	Epoxy	0.16	2.85	9.29 vol%	Ice-template assembly	37
BNNS, BNF	PDMS	0.21	0.56	10 wt%, 0.4 wt%	Vacuum-assisted infiltration	42
Modified BNNS	Epoxy	0.22	0.636	25 wt%	Simple casting	43
BNNS-MWCNT	Epoxy	0.21	4.18	70 wt%	Simple casting	This work

**Fig. 7:**
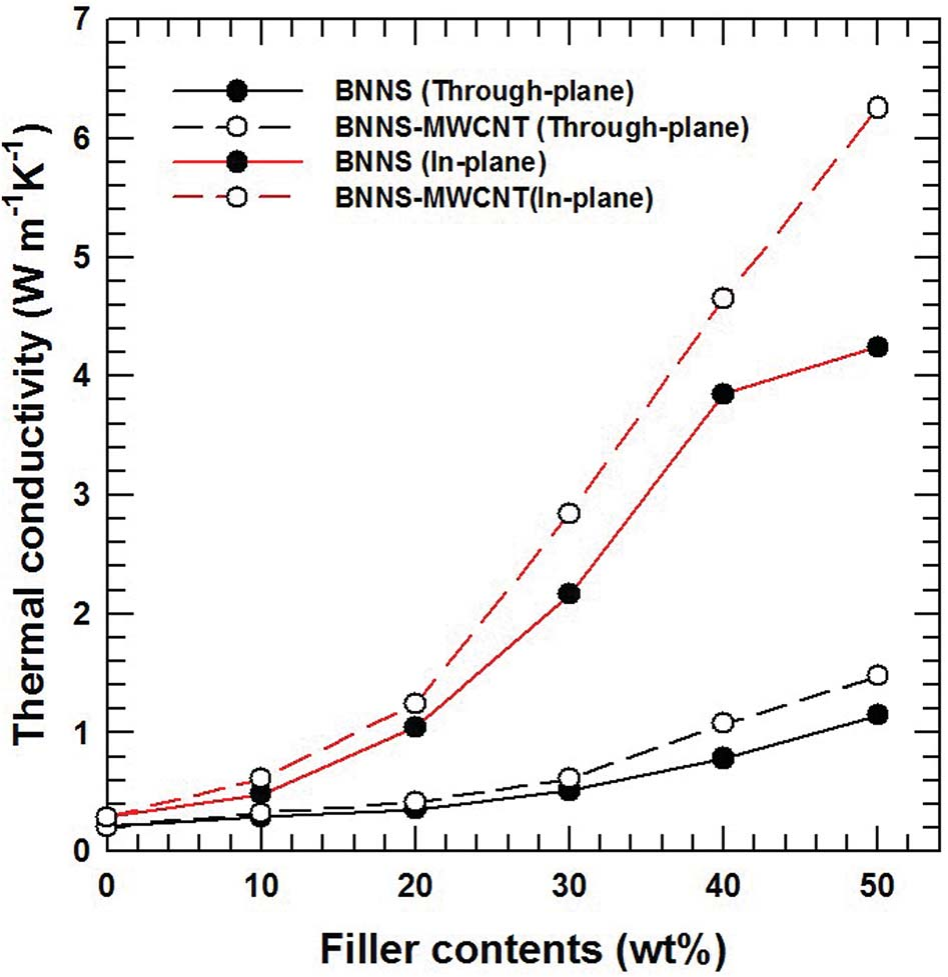
Thermal conductivity of PPS composites with BNNS and BNNS-MWCNT fillers. The black lines are through-plane thermal conductivity, and the red lines are in-plane thermal conductivity.

**Fig. 8:**
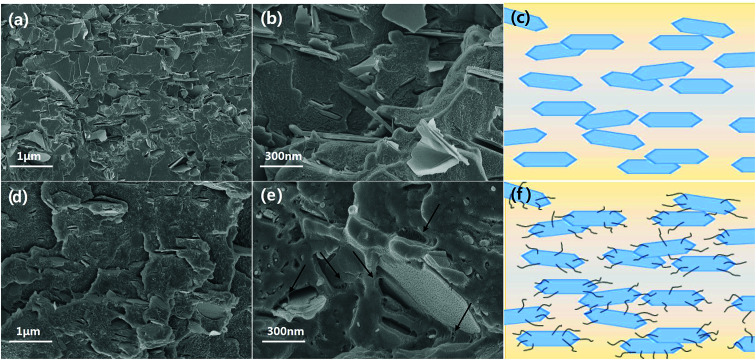
Cross sectional FE-SEM images of composites with 30 wt% filler loading and schematic diagram of PPS composites with BNNS and BNNS-MWCNT fillers. (a–c) BNNS composite, and (d–f) BNNS-MWCNT composite (denoted arrows are MWCNTs).

This hybridization is also effective for a thermoplastic-based composite material. Among the various thermoplastics, PPS-based composites were fabricated using the same filler. Most thermoplastics and their composites are manufactured *via* melt mixing and injection molding. It is well known that anisotropic particles are horizontally oriented and shown anisotropic property as a result of a shear force during injection.^44,45^ In particularly, a BN composite has huge gaps according to the direction because of the high aspect ratio and a relatively low endemic thermal conductivity in the perpendicular direction compared to the horizontal direction.^46,47^ As shown in Fig. 7, the chemical bonding of BNNSs and MWCNTs effectively enhances the both in-plane and through-plane thermal conductivity of PPS-based composites. The FE-SEM images of composites with 30 wt% filler loading and corresponding schematic diagrams in Fig. 8 show that most of the BNNSs are also horizontally oriented *via* a shear force, which almost contributed to the in-plane thermal conductivity. However, the MWCNTs were relatively randomly oriented due to their extremely small particle size and connected or reduced the inter-particle distance of the horizontally oriented BNNSs, which effectively enhances the through-plane thermal conductivity of injection molded composite materials. As a result, chemically bonded MWCNTs on BNNS effectively enhance the thermal conductivity in both direction.

**Fig. 9:**
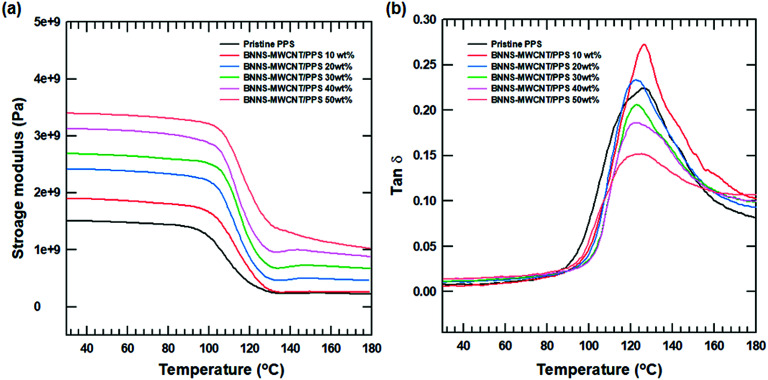
DMA results of BNNS-MWCNT/polymer composite with various filler content. (a) Storage modulus of composite and (b) tan *δ* of composite.

The mechanical and viscoelastic properties of polymer composites was evaluated using dynamic mechanical analysis (DMA) with various BNNS-MWCNT content as a function of temperature. The storage modulus of all the composites tended to increase with increasing amounts of BNNS-MWCNT due to intrinsic hardness and strong modulus.^48^ And as the temperature increased to the glass transition temperature (T0067), the polymer chains became softened and the storage modulus decreased sharply. As shown in Fig. 9(b), the *T*_g_ of the composites increased slightly with increasing filler. However, *T*_g_ of the composites decreased with increasing fillers. This phenomenon was caused by the plasticization effect of the polymer composites.^49^ More than a certain amount of filler decreased the interaction between the polymer chains and gradually decreased the *T*_g_.

## Conclusion

In this study, a chemically connected BNNS/MWCNT hybrid filler was synthesized and applied as a thermal conductive filler. The BNNSs were prepared from bulk BN powder on the tens of micrometers scale *via* exfoliation after heat treatment with vapor in a tubular furnace. The repulsive force from the introduced hydroxyl group exfoliated each BN layer during sonication. The prepared BNNSs were chemically modified using a silane coupling agent. To introduce reactive sites, the MWCNTs were also chemically modified *via* acid treatment. Finally, the BNNS/MWCNT hybrid particles were achieved using a dehydrating agent, DCC. Moreover, two types of fabrication processes were adopted. One was a simple solvent process, and the other was called VFSM. VFSM was the more effective method for chemically bonding the two types of filler because it maximized the particle connections for a sufficient time. As a result, a larger number of MWCNTs were introduced onto the BNNSs. The thermal conductivity of the epoxy-based composite was examined using the fabricated hybrid filler, and the particles fabricated *via* VFSM showed a higher thermal conductivity because of the number of introduced MWCNTs. For a more accurate comparison, the number of MWCNTs was modified by adding MWCNTs. In this case, the VFSM method also had an outstanding result because the particle connections between the BNNSs and MWCNTs were maximized, which generated heat flow paths. Finally, this hybrid particle was compounded with thermoplastic PPS. The in-plane thermal conductivity was almost the same as the BNNS composite, but the through-plane thermal conductivity was significantly improved because the horizontally oriented BNNSs were three-dimensionally connected *via* the MWCNTs.

## Conflicts of interest

There are no conflicts to declare.

## Supplementary Material

RA-008-C8RA05620J-s001
