# Supplementary material for: Fabrication of covalently linked exfoliated boron nitride nanosheet/multi-walled carbon nanotube hybrid particles for thermal conductive composite materials
Source: RSC Adv. 2018 Oct 1;8(58):33506–15. doi: 10.1039/c8ra05620j (PMC9086474; doi:10.1039/c8ra05620j)
Supplement: RA-008-C8RA05620J-s001 [file RA-008-C8RA05620J-s001.pdf]

Electronic Supplementary Material (ESI) for RSC Advances.
This journal is © The Royal Society of Chemistry 2018

Supporting Information:

Fabrication of covalently linked exfoliated boron nitride nanosheet/multi-walled carbon nanotube hybrid particles for thermal conductive composite materials

Kiho Kim, Hyunwoo Oh and Jooheon Kim*

School of Chemical Engineering & Materials Science,

Chung-Ang University, Seoul 06974, Republic of Korea

*Corresponding author: jooheonkim@cau.ac.kr (J. Kim)

1. Figures

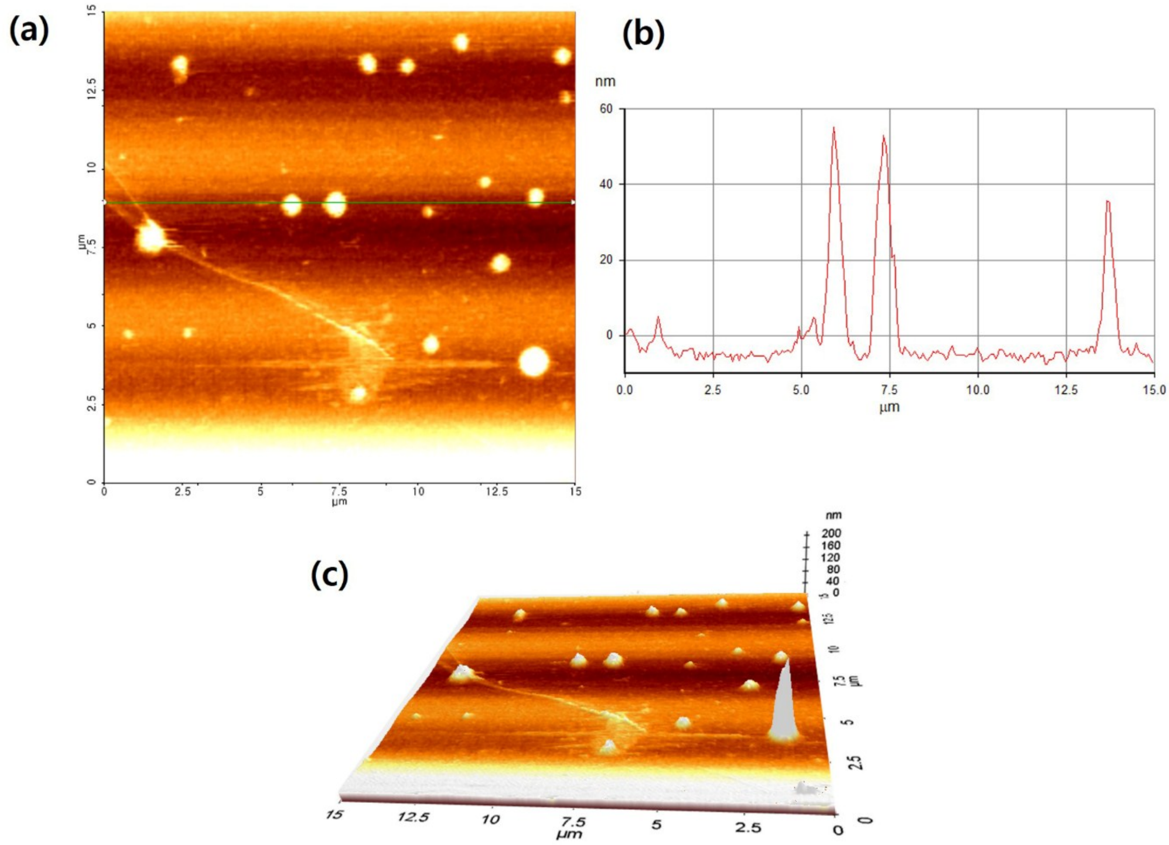

Fig. S1. AFM images of BNNS. (a) 2D AFM image, (b) line profile and (c) 3D AFM image.

We measured the AFM of prepared BNNS to confirm the size and thickness. As shown that the particle size was distributed from 300nm to 1.1 micron and its thickness was 30nm to 80 nm.

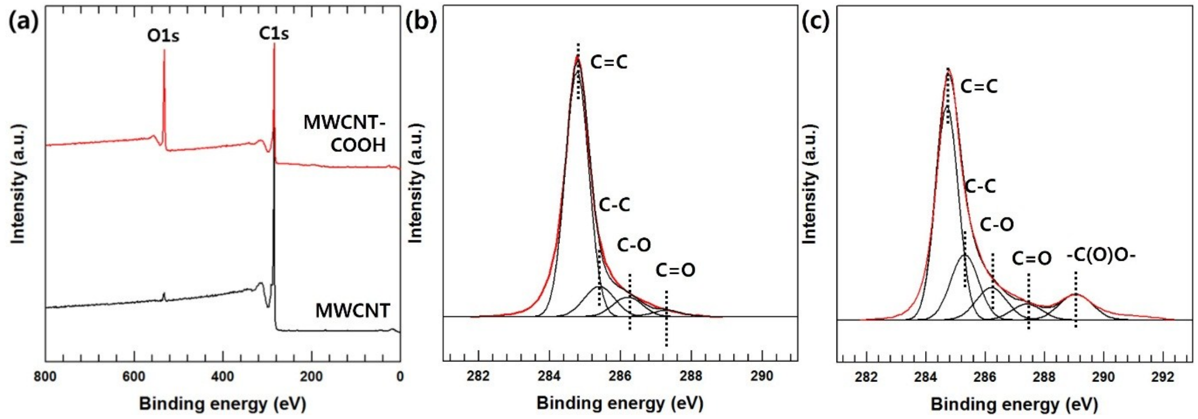

Fig. S2. XPS analysis of MWCNT and MWCNT-COOH. (a) wide scan spectra of MWCNT and MWCNT-COOH, (b) C 1s spectra of MWCNT, and (c) C 1s spectra of MWCNT-COOH.

After the MWCNT acid treatment, the oxygen atom was strongly observed in wide scan spectra. Moreover, the emerged oxgyen functional groups, especially carboxylic group, were strong envidance of functionalization via acid treatment.

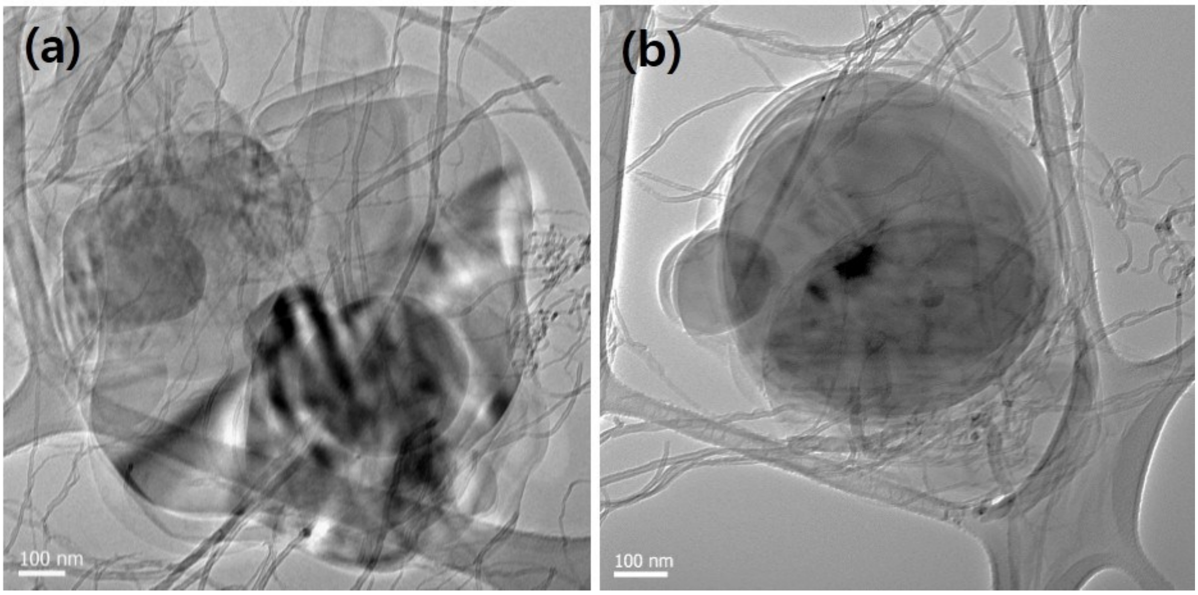

Fig. S3. HR-TEM image of BNNS-MWCNT hybrid particle. (a) fabricated via SP, and (b) fabricated via VFSM)

The MWCNTs were plased on edge of BNNS due to edge functionalization. And, the much amount of MWCNT were observed in fabricaed hybrid particle via VFSM which were corresponed results with FE-SEM and TGA results.

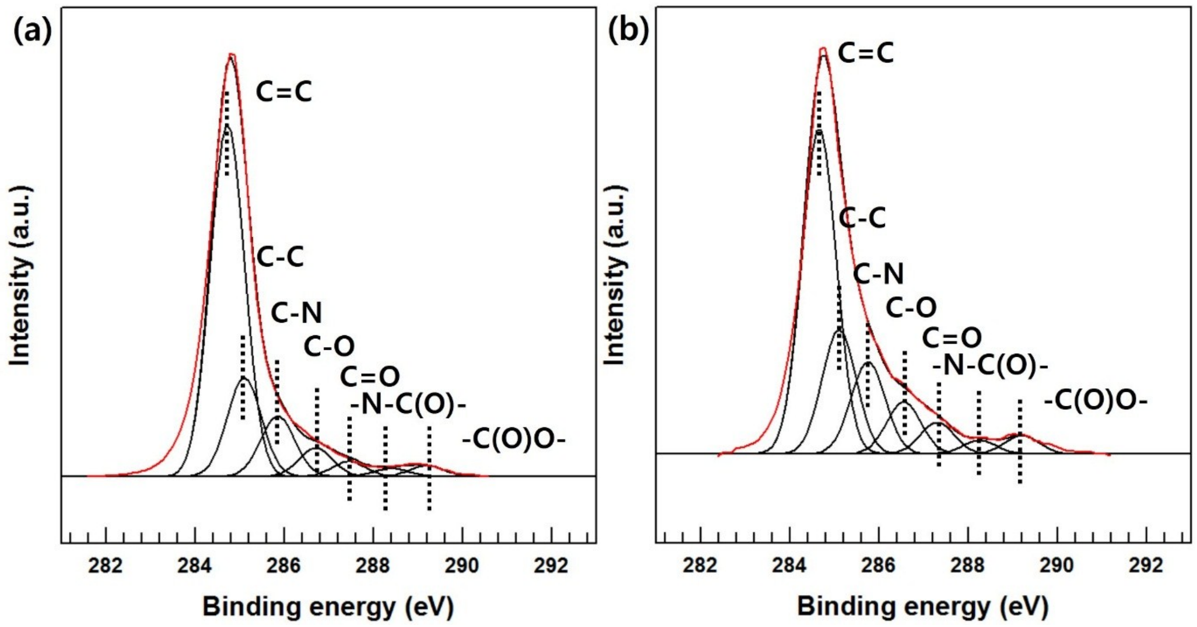

Fig. S4. C1s spectra of BNNS-MWCNT prepared via (a) SP and (b) VFSM.

The both spectra shown similar shape due to chemical structure of two kinds of BNNS-MWCNT was same. However, more strong nitrogen-related functional groups were detected in figure S4(b) due to much amount of MWCNTs were linked to BNNS than another BNNS-MWCNT prepared via SP.
